# A Rare Case of Hereditary Bone Dysplasia: Osteopoikilosis in a Mother and Her Son

**DOI:** 10.7759/cureus.61477

**Published:** 2024-06-01

**Authors:** Bandar A Alghamdi

**Affiliations:** 1 Orthopedic, Department of Surgery, Umm Al-Qura University, Qunfudhah, SAU

**Keywords:** autosomal dominant, benign, lemd3 gene, dysplasia, osteopoikilosis

## Abstract

Osteopoikilosis (OP) is a rare genetic bone dysplasia that causes dense patches in the trabecular bone and occurs in one in 50,000 people. The exact cause is unknown, but it could be due to mutations in the LEM domain-containing gene 3. Two cases were discovered incidentally in a clinic. The first case involved the mother, a 35-year-old woman with type 2 diabetes and dyslipidemia who presented with left ankle and right forearm pain after falling downstairs. Physical examination revealed mild swelling and tenderness at the left ankle, and X-ray examination revealed multiple small sclerotic lesions. The patient was diagnosed with OP. Analgesics, ankle support, and follow-up care were provided. The second case involved the son, a 14-year-old boy who had occasional pain in his right foot. A physical examination was normal. An X-ray of the right foot showed multiple homogeneous sclerotic lesions. He was diagnosed with familial OP and given analgesics for his pain.

## Introduction

Osteopoikilosis (OP) is a rare genetic sclerosing bone dysplasia in which the secondary spongiosa fails to resorb, leading to the formation of dense patches or streaks within the trabecular bone [1]. It is a benign disease originally described by Stieda in 1905 [2]. A more detailed description based on a unique radiographic appearance was created by Albers-Schönberg in 1915 [3]. The disease's incidence has been reported to occur in approximately 1 in every 50,000 individuals [4]. Research has shown that the disease is equally common in both sexes at all ages, but that adult men are more likely to get it than women [5].

The exact underlying mechanisms and etiology of OP are still not fully understood [6]. According to recent research, a mutation of the LEM domain-containing gene 3 (LEMD3) may be the cause of the autosomal dominant pattern of OP [7]. Furthermore, sporadic OP secondary to mutations in the LEMD3 gene has been reported in the literature [3]. The gene LEMD3, which codes for the inner nuclear membrane protein Man1, is crucial for the development of mesenchymal tissue, including the maturation of endochondral bone [8,9]. It is anticipated that the mutated gene product lacks the Man1 Smad-binding domain, which is necessary to inhibit transforming growth factor beta (TGF-β) signaling and bone morphogenic proteins (BMPs). This results in bone sclerosis, which manifests as dense patches on radiographic imaging that resemble enostosis [10-12].

OP should not be confused with other disorders, such as osteoblastic metastases, osteosclerotic myeloma, mastocytosis, and tuberous sclerosis, which also have characteristic radiographic features [6]. These disorders should be considered while making a differential diagnosis. In this study, we report two cases of OP discovered incidentally in our clinic.

## Case presentation

The first case was a 35-year-old female patient who presented to our orthopedic clinic complaining of pain in the left ankle and right forearm that lasted almost 12 hours after falling down the stairs. The pain was a little sharp and worsened when she rotated her right forearm and stood on her left lower extremity. She did not report having any constitutional symptoms. She had a past medical history of type 2 diabetes mellitus and dyslipidemia and was taking oral medications. She had a past surgical history of bariatric surgery. She was otherwise well and had no personal or family history of malignancy.

On physical examination, there was ecchymosis on the dorsal aspect of the right mid-forearm and mild swelling and tenderness on the lateral malleolus of the left ankle. The active and passive range of motion of the left ankle was limited due to pain. The other joints examined were normal. There were no rashes or skin lesions. Other systemic examinations showed no abnormalities.

**Figure 1:**
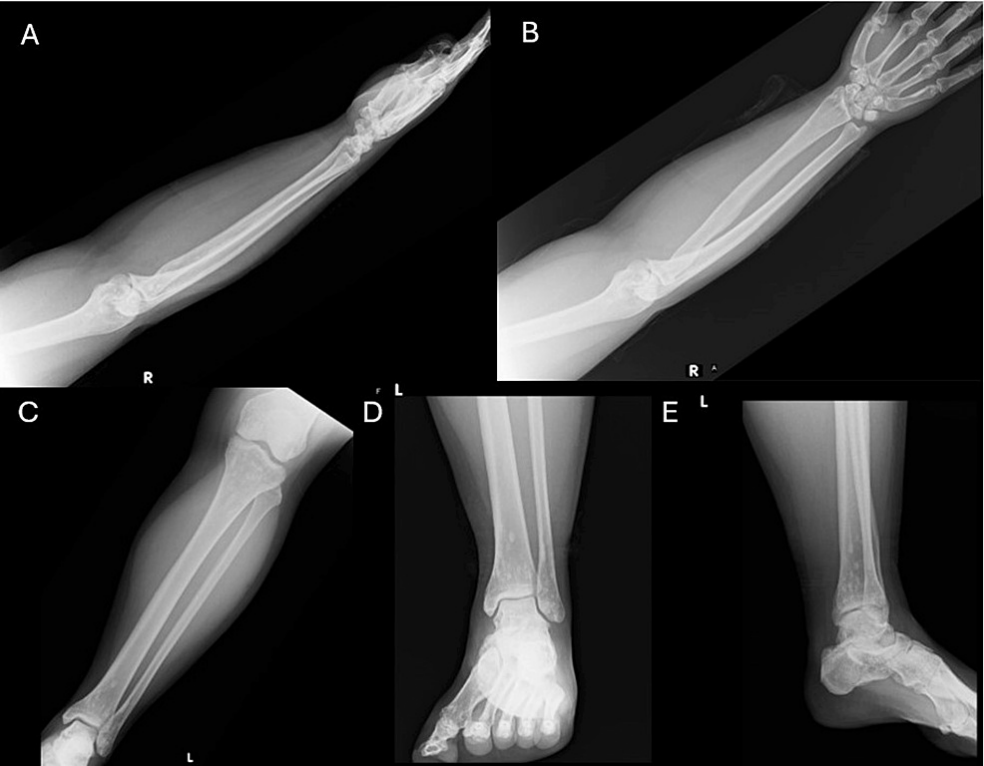
Plain radiographs of the mother, a 35-year-old woman, show several small, well-circumscribed, oval to round sclerotic lesions on the right forearm, left leg, and left ankle, which are distributed in the periarticular regions. (A) Lateral view of right forearm; (B) Anteroposterior view of right forearm; (C) Anteroposterior view of left leg; (D) Anteroposterior view of left ankle; and (E) Lateral view of left ankle.

The radiographic examination of the left ankle and right forearm revealed several small, well-circumscribed, oval to round sclerotic lesions that were distributed periarticularly at the elbow, wrist, knee, and ankle (Figures 1A-1E). The cortical regions did not exhibit any fractures, cortical erosion, or periosteal reactions. The results of the laboratory test, full blood count, bone profile blood test, blood chemistry, inflammatory markers, and tumor marker levels were not significant.

After reviewing the unique radiological findings and ruling out any red flags during the assessment, along with insignificant laboratory results, we determined that the patient has OP.

It was explained to the patient about the benign nature of the bone lesions. Analgesics, ankle support, and follow-up were given to her.

Three months later, she returned to our clinic with her 14-year-old son, who had complained of occasional right foot pain for the past month. The pain was mechanical in nature and increased, particularly when walking. He had no past or recent history of trauma or chronic illness and was not taking any medications. 

A general physical examination revealed no gait disturbance. There was no swelling or tenderness on the right foot. The range of motion of the ankle, subtalar, tarsometatarsal, and metatarsophalangeal joints of the right foot was normal. The examination of other joints and the spine was normal. No skin lesions or other anomalies were found. Regular laboratory tests yielded no pathology findings.

**Figure 2:**
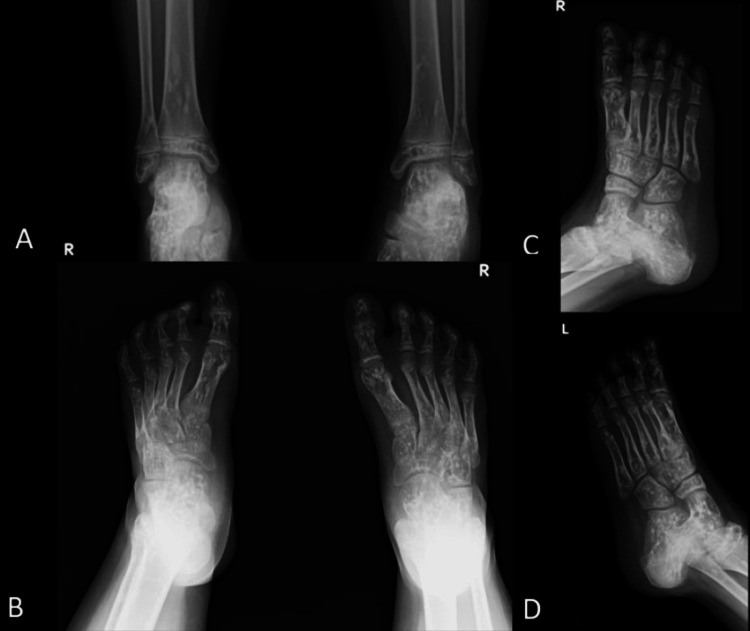
Plain radiographs of the son, a 14-year-old boy, show multiple homogeneous sclerotic lesions on the ankles and feet. (A) Anteroposterior view of ankles; (B) Anteroposterior view of feet; (C) Oblique view of right foot; and (D) Oblique view of left foot.

A plain radiography examination of the right foot in both oblique and anteroposterior views revealed the same feature of OP: multiple homogeneous sclerotic lesions distributed over almost every visible bone system (Figures 2A-2D), leading us to the diagnosis of familial osteopoikilosis. The patient received analgesics as therapy, along with instructions for routine follow-up visits.

## Discussion

OP is a rare inherited disease of endochondral ossification characterized by numerous benign enostoses discovered incidentally on skeletal radiographs [7,12]. There is a tendency within the family that points to an autosomal dominant inheritance [7]. We present two cases in a single family in the current study.

Although the bone lesions are typically asymptomatic, reports indicate that 20% of patients experienced joint symptoms like pain and effusion [3,13,14]. In our second case, there was joint pain. Numerous theories exist to elucidate the mechanisms responsible for joint pain. It may occur incidentally as a result of OP, joint capsule irritation, increased bone metabolism in the vicinity of the bony lesions, or elevated intraosseous pressure as a result of venous stasis at the lesion site [6,13,15].

Conversely, around 25% of patients with OP experience cutaneous manifestations, including dermatofibroma, keloids, or discoid lupus erythematosus. The coexistence of dermatofibroma with OP is known as Buschke-Ollendorff syndrome [7,14,16]. However, we did not detect any dermatological abnormalities in any of our cases.

Remarkably, OP can occur concurrently with other bone dysplasias such as melorheostosis and osteopathia striata, referred to as overlap syndromes [17]. The literature reports that OP is associated with various musculoskeletal conditions, including myelopathy, rheumatoid arthritis, syndactyly, dwarfism, fibromyalgia syndrome, Klippel-Feil syndrome, and spinal canal stenosis. Along with musculoskeletal disorders, other conditions include scleroderma, diabetes mellitus, dental and facial anomalies, scleroderma, dacryocystitis (Gunal-Seber-Basaran syndrome), coarctation of the aorta, urogenital defects, and precocious puberty [3,15,17-20]. Our first patient had previously been diagnosed with diabetes mellitus.

Radiographically, the bone islands of OP are homogeneous and symmetrical, vary in size up to 10 mm in diameter, lie parallel to the surrounding trabeculae, and are usually clustered around joints. Therefore, they are longitudinal, where there are clearly defined linear trabeculae, and spherical, where the linear organization of the trabeculae is less clearly defined [15,21]. Moreover, most lesions are located in the appendicular skeleton and pelvis, while the axial skeleton is largely unaffected. Involvement of the cranial vault is rare [22]. Conversely, osteoblastic metastases are the most prevalent condition, radiologically resembling OP, and can be distinguished by their characteristic asymmetric distribution, size diversity, bone destruction, and periosteal reactions. They are typically found in the axial skeleton, particularly the spine [23]. In our cases, the diagnosis of OP was established based on the patient's history, clinical manifestations, and radiographic findings; therefore, we did not carry out additional testing.

OP is one of the “do not touch” lesions in the skeleton, which means further diagnostic tests or invasive procedures such as a biopsy are unnecessary and may be harmful to the patient [24,25]. Education and reassuring patients are crucial elements of treatment [26]. In the case of joint pain, as in our second case, anti-inflammatory drugs and analgesics can be used to relieve pain [27].

Although the risk of malignant transformation from OP is very low, cases of complications, including giant cell tumors, chondrosarcoma, and osteosarcoma, have been reported; however, no obvious correlation has been found [15]. As a result, it is advised that patients with OP have routine follow-up.

## Conclusions

OP is a rare benign bone dysplasia. However, two cases were discovered incidentally in the same family. Diagnosis is based on typical radiological lesions and normal laboratory tests, as well as the absence of red flags on clinical assessment. It is important to distinguish OP from osteoblastic metastases to prevent unnecessary investigation and invasive procedures and to reduce patient anxiety and fear. Due to the possible risk of malignant degeneration, follow-up examinations of patients are therefore a must.
